# Aggregated Power Indices for Measuring Indirect Control in Complex Corporate Networks with Float Shareholders

**DOI:** 10.3390/e25030429

**Published:** 2023-02-27

**Authors:** Izabella Stach, Jacek Mercik, Cesarino Bertini, Barbara Gładysz, Jochen Staudacher

**Affiliations:** 1 Faculty of Management, AGH University of Science and Technology, Al. Mickiewicza 30, 30-059 Kraków, Poland; 2 Faculty of Finance and Management, WSB University in Wrocław, Fabryczna 29-31, 53-609 Wrocław, Poland; 3 Department of Management, University of Bergamo, Via dei Caniana 2, 24127 Bergamo, Italy; 4 Department of Operations Research and Business Intelligence, Faculty of Management, Wrocław University of Science and Technology, Wybrzeże Wyspiańskiego 27, 50-370 Wrocław, Poland; 5 Fakultät Informatik, Kempten University, Bahnhofstr. 61, 87435 Kempten, Germany

**Keywords:** cooperative game theory, corporate shareholding networks, direct and indirect control, power indices, *Z*-fuzzy number

## Abstract

The purpose of this paper is to introduce new methods to measure the indirect control power of firms in complex corporate shareholding structures using the concept of power indices from cooperative game theory. The proposed measures vary in desirable properties satisfied, as well as in the bargaining models of power indices used to construct them. Hence, they can be used to produce different pictures of the coalitional strength of firms in control of other firms in mutual shareholding networks with the presence of cycles. Precisely, in the framework of Karos and Peters from 2015, ten power indices substitute the original Shapley and Shubik power index in a modular fashion. In this way, we obtain a set of new measures called aggregated indices. The float shareholders typically hold less than 5 percent of the outstanding shares, which is an uncertain element of indirect control in complex shareholding structures. The fuzzy number seems appropriate to model these shareholders’ behavior. The novelty is that we model the behavior of float using *Z*-fuzzy numbers. The new methods are tested in an example.

## 1. Introduction

The measurement of indirect control in complex shareholding structures is an important element of the financial analysis of such structures. Such analysis can be used for various purposes by management boards of companies that are elements of corporate networks, for takeovers, or to have sufficient control to influence decisions of a particular firm, for example. The novel proposal of this paper is to offer a differentiated set of measures of indirect control. We obtain these measures by modifying the approach of Karos and Peters (2015) [1]. In particular, our method applies ten power indices substituting the original Shapley and Shubik (1954) [2] power index in a modular fashion. The ten considered power indices differ significantly with regard to, for example, the known and desirable properties in simple games. They highlight different aspects of the voting situation. We called our indirect control measures the aggregated power indices due to the formula used in the Karos and Peters approach. 

One of the interesting issues regarding modeling the complex shareholding networks relate to the presence of float—the set of unidentified shareholders, frequently with less than 3% or 5% of shares of a company—in the network. Some authors disregard the float altogether, and others try to model it; see Crama Leruth (2013) [3] for a review of the literature on this question. In this paper, we also try to touch on this issue and calculate some aggregated indices considering the float in a corporate network in an example. Note that Karos and Peters, when introducing their model in [1], used the example of the Porsche and Volkswagen shareholding network and disregarded the float. Their example was based on the annual reports 2012 of Volkswagen AG and Porsche Automobil Holding SE GmbH. For the Volkswagen AG company, they omitted the float of small shareholders (2.2%) and combined slightly larger shareholders into one monolith—others (9.9%).

When preparing an analysis of a company being a part of a shareholding structure, one has to pay attention to two (among other) important factors:

1. The size of shareholding does not reflect the degree of shareholder control power over corporate matters. In other words, a shareholder can have substantially more or less corporate control than the shareholding percentage may suggest. Consider the example of a company with three shareholders: the first shareholder owns 70% of the shares, the second shareholder owns 20%, and the third shareholder owns the remaining 10% of the shares. If only shareholding size is examined, it would appear that the degree of control for each of the three investors is not in proportion to the percentage of their shareholdings, as the shareholder with 70% of shares controls this company fully. The situation may be even more complicated when the so-called right to block a majority coalition exists (the right of veto, which is established for other than economic reasons mostly). For example, in Poland, there is the concept of the so-called “golden share”, the possession of which allows you to block some decisions of the majority coalition. In the Volkswagen AG company, which is the basis of the example analyzed in [1], this role is played by a “4/5 rule”, which gives the State of Lower Saxony a blocking majority, as it controls more than 20% of the shares.
2. There exists indirect control over companies when, for example, one fully controlled company is a majority shareholder of another company.

These two aspects justify why we use the power indices—concepts of solution from the cooperative game theory—in our work and how important it is to measure indirect control in complex shareholding structures. The game theory approach to measuring the indirect control power of firms as elements of a whole corporate network is known in the literature on the subject. Many scholars proposed methods based on power indices for measuring the indirect control power of a firm in an ownership network; see Gambarelli and Owen (1994) [4], Turnovec (1999) [5], Hu and Shapley (2003) [6,7], Leech (2002) [8], Crama and Leruth (2007, 2013) [3,9], Karos and Peters (2015) [1], Mercik and Lobos (2016) [10], Levy and Szafarz (2017) [11], Mercik and Stach (2018) [12], Stach, Mercik, and Bertini (2020) [13], Staudacher, Olsson, and Stach (2021) [14], Stach and Mercik (2021) [15], Staudacher, Olsson, and Stach (2022) [16], and Stach, Mercik, and Bertini (2023) [17], for examples. The reader can find the comparisons of some of these approaches in [18,19,20] and [12]. Let us also not forget that Penrose (1946) [21] and Shapley and Shubik (1954) [2] were the first to point to this area of application of power indices.

**Figure 1:**
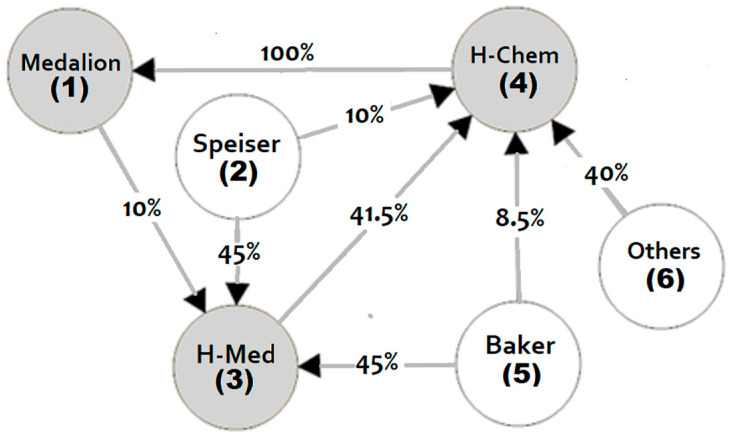
The shareholding network of the Speiser–Baker case. H-Med = HealthMed, H-Chem = HealthChem. Source: [10,18].

A complex corporate shareholding network is a network separated from a given market of companies, investors, and float with cross-holdings and cycle ownership. An example can be presented by a directed graph, as illustrated in Figure 1. The Karos and Peters approach takes all firms in measuring the indirect control and has no problem with cycle-ownerships. It should be highlighted that only a few of the methods mentioned above in the literature have this characteristic [1,10,11,12,13,14,15]. It is also a justification for our choice of method to focus.

In practice, it is often very hard to obtain all the data on all firms in an ownership structure, especially if they are not quoted on the stock market. As the float can impact the control structure and control power of companies, it is also a good idea to include it in the model. In this paper, we present two methods to incorporate the float. One relatively simple approach is motivated by a result by Dubey and Shapley (1979) [22]; see Section 3.5. The second one uses fuzzy numbers; see Section 4.

In the literature, authors consider Shapley and Shubik’s [2] index in cases when the weights of players are uncertain, and that uncertainty is modeled by fuzzy weights. Elena Mielcová (2016) [23] proposes the concept of the Shapley and Shubik index voting power under intuitionistic fuzzy sets. In the work [24], the Shapley and Shubik index is considered for the description of a voting game in parliamentary voting. A fuzzy coalition is a vector with coordinates called the membership degrees of a player in a coalition. The membership of such a player in a coalition is the probability of the political party’s occurrence in the coalition. Przybyła-Kasperek (2021) [25] considers the Shapley–Shubik index in cases in which global decisions are taken based on local decisions. The rough set theory proposed by Pawlak (1985) [26] is used to construct the membership function of the player/agent. In this article, we propose using the *Z*-fuzzy number to model uncertainty in players’ weights.

The remainder of the paper is structured as follows. Section 2 gives the formal notations and definitions of simple and weighted games, power indices, and the desirable properties of power indices in simple games. In particular, in Section 2.2.2, the reader can find the formulas of power indices used to build the aggregated power indices. Section 2.3 presents the Karos and Peters approach. In Section 3, we define the set of aggregated indices and illustrate them in an example of a corporate shareholding network. Still, in this section, we extend the Karos and Peters approach considering the binary float of small shareholdings (Section 3.5). The fuzzy float in the Karos and Peters approach is regarded in Section 4. Section 5 contains some discussions, concluding remarks, and further developments.

## 2. Notation and Definitions

Any complex corporate shareholding network, with cross-holdings and cycle ownership, is presented by a directed graph where nodes represent shareholders (companies, investors, and float) and arcs represent relationships between them. 

### 2.1. Simple and Weighted Games

Let *N* = {1, …, *n*} be a finite set of *n* players and 2*^N^* be the set of all subsets of *N*. Any element of 2*^N^* is called a coalition. In particular, *N* is called a grand coalition and ∅ is an empty coalition. A simple *n*-person game is a pair (*N*, *v*), where $v:2^{N}⟶\left\{0,1\right\}$, is a real-mumber characteristic function satisfying the following conditions:

- value of an empty coalition is equal to zero: v(∅)=0;
- value of a grand coalition is equal to 1: *v*(*N*) = 1;
- (monotonicity) v(S)≤v(T) for all coalitions *S* and *T,* such that S⊆T⊆N.

A coalition $S\in 2^{N}$ is a winning one if v(S)=1; otherwise, (v(S)=0) it is said to be a losing coalition. Let *W* denote the set of all winning coalitions in a simple game (*N*, *v*). A simple game is said to be proper if ∀S⊆N if v(S)=1; then, v(N\S)=0. In this paper, we analyze only proper simple games (for more of a proper simple game, see (Stach 2011) [27]). A player *i* is said to be critical in coalition *S* if *v*(*S*) = 1 and v(S\{i})=0. The set of all winning coalitions in a simple game (*N*, *v*), in which *I* is critical, is denoted by $\eta_{i}(v)$. For each coalition, $S\in 2^{N}$ *Cr*(*S*) denotes the number of critical players in *S*, which means the number of players whose deletion from *S* is critical. A winning coalition *S* is said to be a minimal winning coalition if *Cr*(*S*) = |*S*|, where |*S*| stands for the size/cardinality of *S*, i.e., the number of members of *S*. *W^m^* stands for the set of all minimal winning coalitions in a simple game (*N*, *v*). *W^ls^* stands for the set of all winning coalitions of the least size in the game (*N*, *v*). A winning coalition of the least size is obviously a minimal winning coalition, so $W^{ls}\subseteq W^{m}\subseteq W$. A winning coalition is called vulnerable if at least one of its members is critical (we also say that this player is in a swing position, i.e., a change in that member’s vote to “no” would cause the coalition to lose). If only one player is critical, then this player is uniquely powerful in the coalition. The Inverse of the number of critical players is called the fractional swing for coalition S, *FS*(*S*) = 1/*Cr*(*S*). For example, if there are two critical players in coalition *S*, then *FS*(*S*) = 1/2. Let *VC* denote the set of all vulnerable coalitions.

A player i∈N is said to be a null player if the following equation holds for each coalition $S\in 2^{N}$ containing player *i*: v(S)=v(S\{i}). A winning coalition *S* is said to be a null player-free winning coalition if all of its members are non-null players. Following the notation used in [28], $W^{n-}$ stands for the set of all null player free-winning coalitions.

For the sets *W, W^m^*, *W^ls^*, and $W^{n-}$ in a simple game (*N*, *v*), *W_i_,* $W_{i}^{m}$*,*$W_{i}^{ls}$, and $W_{i}^{n-}$ denote the corresponding subsets of *W*, formed by coalitions that contain player *i*. Any simple game may be unequivocally determined by *W*, *W^m^*, and $W^{n-}$, (see Álvarez-Mozos et al. (2015) [29], Stach (2022) [30], and Stach and Bertini (2021) [28]. Thus, in any simple game (*N*, *v*), the set of winning coalitions *W* can always be described by the set of null player-free winning coalitions $W^{n-}$ as follows: $W=\{S\in 2^{N}:\;\exists T\in W^{n-}|T\subseteq S\}$.

Isbell (1958) [31] introduced the so-called (*strict*) *desirability* relation. In a simple game (*N*, *v*), player *i* is said to be (*strictly*) *more desirable* than *j*, denoted by i≻j if the following two conditions are satisfied:

- ∀S⊂N, such that i∉S and j∉S, S∪{j}∈W⇒S∪{i}∈W.
- ∃T⊂N, such that i∉T and j∉T, T∪{i}∈W and T∪{j}∉W.

If for a pair of players i,j∈N and each coalition $S\subseteq N\backslash \left\{i,j\right\}$, the following biconditional statement is true: $S\cup \left\{i\right\}\in W\;↔\;S\cup \left\{j\right\}\in W$. Then, players *i* and *j* are said to be equally desirable, denoted by i∽j. Next, player *i* is at least desirable as *j* if i≻j or i∽j, denoted by i≿j.

The *desirability* relation (≿) suggests that the more desirable a player is, the more powerful s/he should be. A minimal winning coalition *S* is *shift minimal* if for each member i∈S and j∉S, such that i≻j, it holds (S\{i})∪{j}∉W. This implies that in a shift minimal coalition, there are no surplus players, and a weaker player can replace no player according to the strict desirability relationship without altering the winning status of the coalition. Using $W^{sm}$, we denote the set of all shift minimal coalitions in a simple game. Then, using $W_{i}^{sm}$ we denote the corresponding subsets of *W*, formed by coalitions that contain player *i*. 

A simple game (*N*, *v*) is said to be complete (or linear or swap robust) if the desirability relation is a complete preordering. Generally, it means that for each pair of players, we can express who is more desirable: ∀i,j∈N i≻j, or j≻i (or both).

A proper simple game (*N, v*) is said to be a weighted game—denoted by $\left[q;w_{1},\ldots ,w_{n}\right]$—if there exist non-negative real numbers, $w_{1},w_{2},\;\ldots ,\;w_{n}$, such that for every coalition, S⊆N, S∈W if and only if the sum of *w*_*i*_’s, i∈S, is at least equal to *q*. The number $w_{i}\geq 0$ stands for the weight of player *i* and a non-negative quota *q* is called the decision rule, i.e., the minimum amount of weights necessary to pass a decision. Usually, $\frac{1}{2}\sum_{i=1}^{n}w_{i}<q\leq \sum_{i=1}^{n}w_{i}$.

Since in each weighted game, the players can be arranged in the order determined by non-decreasing weights, each weighted game is complete as well. So, weighted games have this property that players are ordered by the desirability relationship.

### 2.2. Power Indices

A power index *f* is a function mapping a unique vector $f(v)=(f_{1}(v),\ldots ,f_{n}(v))$ to every simple game (*N*, *v*). Many power indices have been proposed after the most known and widely applied Shapley and Shubik (1954) [2] power index. In general, power indices are used to measure the power of a player *i* in group decision-making bodies. However, the interpretation of this value—$f_{i}\left(v\right)$—assigned to player *i* changes and depends also on the measure (power index) itself and the context in which it is applied, see Felsenthal and Machover (2005) [32], Laruelle et al. (2006) [33], Gambarelli and Stach (2009) [34], Bertini and Stach (2011) [35], Bertini et al. (2013) [36], Bertini et al. (2015) [37], Bertini and Stach (2015) [38], Stach (2016) [39], Bertini et al. (2016) [40], Bertini et al. (2017) [41], Bertini et al. (2018) [42], Bertini et al. (2020) [43], Staudacher et al. (2021) [44], Stach and Bertini (2021) [28], Stach (2022) [30], Stach and Bertini (2022) [45], and Stach, Mercik, and Bertini (2023) [17].

#### 2.2.1. Some Desirable Properties of Power Indices

In the literature on the topic, we can find some desirable properties (also called postulates) of power indices in simple games. We list here only those that we regard suitable for measuring the indirect control of firms in corporate shareholding structures and for the considered framework. Nevertheless, in general, the following postulates are widely accepted. 

Postulate 1 (Anonymity): $f_{i}\left(v\right)=f_{\pi \left(i\right)}\left(\pi \left(v\right)\right)$ for every simple game (*N*, *v*), every player i∈N, and each permutation π:N↦N, where the simple game $\left(N,\;\pi \left(v\right)\right)$ is defined as follows: $\pi v\left(S\right)=v\left(\pi \left(S\right)\right)$ for every coalition S⊆N. The game *(N*, *πv*) is the same as (*N*, *v*), except that players are relabeled according to π. Less formally, this property states that the value assigned by a power index *f* does not depend on the player’s name.

Postulate 2 (Efficiency): $\sum_{i\in N}f_{i}(v)=1$ for every simple game (N,v). This postulate states that the sum of powers assigned by a power index *f* to all players must equal 1 (100%), i.e., the total power in the game.

The three postulates that follow are the original transfer axioms proposed by Dubey [46] to characterize the Shapley [47] value and its two equivalent variants. The idea behind presenting the two variants is to better understand what the transfer postulate requires from a power index based on winning or minimal winning coalitions. 

To state the next postulate, let us introduce the following notation. For all pairs of simple games, (*N*, *v*) and (*N*, *w*), let us define (v∧w) and (v∨w) by the following sets of winning coalitions: $W(v_{1}\wedge v_{2})$ = $\left\{S\in 2^{N}:S\in W(v_{1})\;\mathrm{and}\;S\in W(v_{2})\right\}$, $W(v_{1}\vee v_{2})$ = $\left\{S\in 2^{N}:S\in W\left(v_{1}\right),\;\mathrm{or}\;S\in W(v_{2})\right\}$. Note that the set of all simple games is closed under operations ∧, ∨. Thus, a coalition is winning in (N,v∨w) if, and only if, it is winning in at least one of *v* or *w*, and it is winning in (N,v∧w) if, and only if, it is winning in both (*N*, *v*) and (*N*, *w*). 

Postulate 3 (Transfer—Dubey (1975) [46]): $f_{i}(v\wedge w)+f_{i}(v\vee w)=f_{i}(v)+f_{i}(w)$ for all pairs of simple games (*N*, *v*), (*N*, *w*) and each player i∈N.

Postuale 3′. (Transfer—Dubey and Shapley (1979) [22]): consider two pairs of simple games, (*N*, *v*) and (*N*, *v*′) and (*N*, *w*) and (*N*, *w*′), and suppose that the transitions from (*N*, *v*′) to (*N*, *v*) and (*N*, *w*) to (*N*, *w*′) entail adding the same set of winning coalitions, i.e., $W(v)\subset W(v{}^{\prime })$, $W(w)\subset W(w{}^{\prime })$, and $W(v)\backslash W(v{}^{\prime })=W(w)\backslash W(w{}^{\prime })$. Then, the equivalent transfer axiom stats $f_{i}(v)-f_{i}(v{}^{\prime })=f_{i}(w)-f_{i}(w{}^{\prime })$ for each player i∈N. This means that the change in power depends only on the change in the voting game (i.e., on the set of the new winning coalitions). 

Postulate 3″. (Transfer—Laruelle and Valenciano (2001) [48]). For all pairs of simple games, (*N*, *v*) and (*N*, *w*) and all $S\in W^{m}(v)\cap W^{m}(w)$, such that S≠N the following holds: $f_{i}(v)-f_{i}(v_{S}^{*})=f_{i}(w)-f_{i}(w_{S}^{*})$ for all i∈N. 

$\left(N,v_{S}^{*}\right)$ and $\left(N,w_{S}^{*}\right)$ are modified games, such that $W(v_{S}^{*})=W(v)\backslash S$ and $W(w_{S}^{*})=W(w)\backslash S$. This axiom is equivalent to the transfer axiom. In other words—citing Laruelle and Valenciano—this reformulated transfer axiom states that the effect (gain or loss) on any player’s power of eliminating a single minimal winning coalition from the set of winning ones is the same in any game in which this coalition is minimal winning.

Postulate 4. (Null player). If i∈N and *i* is a null player in (*N*, *v*), i.e., *v*(*S*∪{*i*}) = *v*(*S*) for every *S* ⊂ *N*\{*i*}, then $f_{i}(v)=0$. This postulate requires that a player who does not contribute to a coalition should obtain null power.

Postulate 5. (The null player’s removable property). $f_{i}\left(v^{{}^{\prime }}\right)=f_{i}\left(v\right)$ for each simple game (*N*′, *v*′) arising from (*N*, *v*) by eliminating the null players and each non-null player $i\in N^{{}^{\prime }}$, i.e., $i\notin N\backslash N^{{}^{\prime }}$.

Postulate 6. (Local monotonicity, LM). LM requires that a voter *i* who controls a larger share of the vote cannot have a smaller share of power than a voter *j* with a smaller voting weight.

#### 2.2.2. Power Indices Considered in This Paper

Considering a specific property of power indices in simple games, we can divide the indices into certain groups, which are not necessarily disjointed. Let us consider three groups of power indices.

- A group of power indices that satisfy the transfer property—also called the additivity property. To this group belong indices which follow Shapley and Shubik [2], absolute Banzhaf [21,49], Rae [50], Nevison [51], and Solidarity [45,52] indices. From this group of indices, we have chosen only one—the Solidarity index—for developing a group of power indices meant to represent the real power of the firms in a mutually complex shareholding network.
- A group of power indices that are based on minimal winning coalitions. These kinds of power indices were introduced by Deegan and Packel [53], Holler [54,55], Alonso-Meijide and Freixas [56], Alonso-Meijide, Freixas, and Molinero [57], and Felsenthal [58]. We take all indices from this group for further consideration, although this group is the most sensitive considering the postulate of local monotonicity. Namely, in this group of indices, only the *PI* index satisfies this property; see Felsenthal (2016) [58].
- A group of power indices that satisfy the null player removable property. In this group, we have all power indices from the previous group that relate to the minimal winning coalitions. The indices that are based on null player-free winning coalitions proposed by Álvarez-Mozos et al. [29], and the indices proposed by Banzhaf [49], Johnston [59], and Shapley and Shubik [2]. We take all these indices into consideration as well. Note that all indices in this group satisfy the null player property as well.

In the literature, we can find more interesting properties that can be regarded in the context of measuring indirect control. None of the power indices considered by us would satisfy all possible properties. For example, Felsenthal in [58] regarded six properties of the so-called P-power indices, and even the Shapley and Shubik power index failed to fulfill one of them. However, not only the number of compelling properties fulfilled by a power index is important, but also the normative bargaining model underlying this index needs to be convincing.

In the following, we define the power indices considered in this paper. All these indices satisfy the anonymity and efficiency postulates often present in the axiomatic characterization of the following indices if an index has such characterization. The definitions of the power indices below are given for each simple game $\left(N,\;v\right)$ and each player i∈N.

- The Shapley and Shubik [2] power index is defined as follows:

$$\sigma_{i}(v)=\sum_{S\in \eta_{i}(v)}\frac{(n-|S|)!(|S|-1)!}{n!}$$

- The normalized Banzhaf [21,49] power index is defined as follows:

$$\beta_{i}(v)=\frac{\left|\eta_{i}(v)\right|}{\sum_{j\in N}\left|\eta_{j}(v)\right|}$$

- The Solidarity index as a restriction of the Nowak and Radzik (1994) [52] Solidarity value in a simple game (*N*, *v*), see Stach and Bertini [45]. This index is expressed as follows:

$$\psi_{i}\left(v\right)=\sum_{T\subseteq N,i\in T}\frac{1}{\left(\begin{array}{c} n\\ \left|T\right| \end{array}\right)}\frac{Cr(T)}{|T{|}^{2}}$$

- The Deegan and Packel [53] power index is given as follows:

$$\Delta_{i}(v)=\frac{1}{\left|W^{m}\right|}\sum_{S\in W_{i}^{m}}\frac{1}{\left|S\right|}$$

- The Holler index *h* (called also the Public Good Index) [54,55] is given by:

$$h_{i}(v)=\frac{\left|W_{i}^{m}\right|}{\sum_{j\in N}\left|W_{j}^{m}\right|}$$

- The Shift index [56] is defined as follows:

$$s_{i}(v)=\frac{\left|W_{i}^{sm}\right|}{\sum_{j\in N}\left|W_{j}^{sm}\right|}$$

The *s* index can be seen as a modification of the Holler index. The only difference is that it considers the subset of minimal winning coalitions called the shift minimal winning coalitions instead of minimal winning coalitions.

- The Shift Deegan–Packel index [57] is defined as follows:

$$\mu_{i}\left(v\right)=\frac{1}{\left|W^{sm}\right|}\;\sum_{S\in W_{i}^{sm}}\frac{1}{\left|S\right|}$$

The *µ* index combines the ideas of the Shift [56] and the Deegan and Packel [53] indices.

- The Felsenthal [58] power index *PI* of the winning coalitions of least size—proposed by Felsenthal (2016)—can be seen as a slight modification of the Deegan and Packel [53] index by replacing in its underlying assumptions of the minimal winning coalitions by the winning coalitions of least size (WCLS). The Felsenthal power index of WCLS for any player *i*, originally denoted by *PI*, is obtained as follows:

$$PI_{i}\left(v\right)=\frac{1}{\left|W^{ls}\right|}\sum_{S\in W_{i}^{ls}}\frac{1}{\left|S\right|}$$

- The *f^n−^* power index [29] is defined as follows:

$$f_{i}^{n-}(v)=\frac{1}{\left|W^{n-}\right|}\sum_{S\in W_{i}^{n-}}\frac{1}{\left|S\right|}$$

The *f^n−^* index can be seen as a modification of the Deegan and Packel [53] index. The only difference is that *f^n^* considers all null player-free winning coalitions.

- The Álvarez-Mozos et al. [29] *g^n−^* power index is defined as follows:

$$g_{i}^{n-}(v)=\frac{\left|W_{i}^{n-}\right|}{\sum_{j\in N}\left|W_{j}^{n-}\right|}$$

The *g^n−^* index can be seen as a modification of the Holler [54,55] index—with the only difference being that g*^n^* considers all winning coalitions that do not contain null players, or as a restriction of the Public Help Index *θ* [30,60], which is based on all winning coalitions.

- The Johnston [59] index is defined as follows:
  $$\gamma_{i}(v)=\frac{\sum_{S\in VC,i\in S}FS_{i}(S)}{\sum_{j\in N}\sum_{S\in VC,j\in S}FS_{j}(S)}$$
  where for each vulnerable coalition, S∈VC and critical player $i\in S\;FS_{i}\left(S\right)=FS\left(S\right)$, and for non-critical players, $FS_{i}\left(S\right)\;=0$.

### 2.3. The Karos and Peters Approach

Karos and Peters [1] model the indirect control power of agents in a mutual control structure with N firms—such as a corporate shareholding network, for example—in two equivalent ways. The first way is by so-called invariant mutual control structure (*C*)—a map that assigns to each coalition the set of controlled firms in a network. In simple words, an invariant control structure must consider all indirect control relations. The second way is by a simple game structure $v^{C}=\left(v_{1}^{C},\;v_{2}^{C},\;\ldots ,\;v_{n}^{C}\right)$, in which for each firm-player i∈N, a simple game $v_{i}^{C}$ describes who controls that firm. This approach is similar to command games proposed by Hu and Shapley (2003) [6,7].

The Karos and Peters [1] method of measuring indirect control in complex shareholding structures is axiomatic. Karos and Peters based their approach on the five axioms obtaining a unique index Φ. They regarded the following axioms.

**Axiom 1.**  *(Also known as the null player axiom.) The power of each null firm is equal to zero. A firm i is null if is not controlled by any firm and i does not exert any control over other firms in the network.*

**Axiom 2.**  *(Also known as the constant sum property.) The sum of all assigned powers is the same over all invariant mutual control structures based on N. From axioms 1 and 2, it follows that this sum is equal to zero.*

**Axiom 3.**  *(Also known as the anonymity axiom.) The names of the firms should not matter.*

**Axiom 4.**  *(Also known as the transfer axiom.) For any firm, the change in power when enlarging a mutual control structure P to P′ should be equal to the change in power when enlarging a mutual control structure Q to Q′, under the assumption that the same control relations are added going from P to P′ as when going from Q to Q′. This axiom relates to the transfer axiom used to characterize the Shapley value and the Shapley and Shubik index* [2,46,47].

**Axiom 5.**  *(Also known as the controlled player axiom.) If company i is controlled by at least one coalition and, as a consequence, by a grand coalition N, but does not control any company, then the power of company i is set at −1. Next, if a firm j is controlled by no coalition at all, but firms i and j exert the same marginal control with respect to any coalition, then firm j obtains one more than firm i.*

Let $\bar{C}$ be the set of all invariant mutual control structures on *N*. For every i∈N and $C\in \bar{C}$, the Karos and Peters Φ index is defined as follows:

(1) $$\Phi_{i}\left(C\right)=\sum_{k\in N}\sigma_{i}\left(v_{k}^{C}\right)\;-v_{k}^{C}\left(N\right)$$

where $v_{k}^{C}\left(S\right)=1$ if *k* is controlled by *S*; otherwise, $v_{k}^{C}\left(S\right)=0$.

Note that the simple games $v_{k}^{C}\left(S\right)$ are determined by the sets of minimal winning coalitions in direct and indirect control. We recall that a simple game is uniquely determined by the set of its minimal winning coalitions; see Section 2.1. Here, we give the abbreviate definition of the Karos and Peters approach. For details and how to incorporate the indirect control relationships to obtain the invariant mutual control structure—and the so-called minimal winning coalitions that consider direct and indirect control—we cross-refer the reader to [1,61].

## 3. A General Framework of Aggregated Power Indices for Indirect Control

### 3.1. Modelling of Corporate Shareholding Networks

In this work, we consider the approach by Karos and Peters [1] and modify it by substituting the original Shapley and Shubik index with ten others to obtain so-called aggregated power indices. This idea has been just used by Stach, Bertini, and Mercik (2023) [17] and the Holler [54,55] index—also called the Public Good Index—substituted the Shapley and Shubik index in the Karos and Peters framework. The new index obtained was called the *iPGI* index. Nonetheless, to have a total set of aggregated indices in one paper, we dedicate it to *iPGI* in Section 3.4.4.

The choice of the power indices to apply in Karos and Peters’ approach is based on some properties fulfilled by these indices; see Section 2.2.2. Let us try below to justify our choices.

The first group refers to the transfer property, as this property constitutes the fourth axiom of the Karos and Peters index Φ; see Section 3.3. Scarcely few efficient power indices satisfy this property. One of them, and different from the Shapley and Shubik index, is the Solidarity index [45,52]. One can be surprised by the use of this index in the context of measuring the indirect control power of firms in mutual corporate shareholding networks. However, in certain situations, the a priori estimation of power given by the Solidarity index can produce useful information. For example, firms can try to be solidary with other shareholders of the same company to maintain control against a potential hostile takeover. In this context, it could be interesting to find a better and safer redistribution of shares based on the distribution of the solidarity power index or its aggregated version. However, this reverse problem and index could be a subject for future studies. 

The second group contains the power indices based on minimal winning coalitions. Stach, Bertini, and Mercik (2023) [17] argued widely the validity and incisiveness of the choice of the minimal-winning power indices to assess the real power of players in the context of indirect control. We refer the reader to this work, and here we just mention that in the context of a hostile takeover attack, it seems that a potential rider will look for a minimal winning coalition; not one that is expensive, but one that is stable and winning.

The third group refers to the null player removable property. The importance of this postulate in the context of measuring indirect control was highlighted first in [12] and then in [14]. Moreover, Staudacher et al. (2021) [14] present a bit more particular version of this postulate—the null investor removable property—for mutual corporate shareholding structures. In other words, this property says that after removing the investors whose voting rights cannot transform any losing coalition into a winning one, null investors from a network and the non-null firms’ power measures should remain unchanged. Equivalently, the value of any firm in a corporate shareholding network is unchanged if the network is extended by adding a new null investor. Staudacher et al. [14] noted that the Φ index satisfies the null investor removable property. Moreover, the minimal winning coalitions and Johnston indices satisfy the null player removable property in simple games. So, as a consequence, their aggregated versions must also fulfill the null investor removable property.

As we mentioned in the Section 1, some researchers disregard the presence of float in corporate shareholding networks, and some try to model it, as the power of float can influence the power of big shareholders. Only a few researchers dealing with modeling indirect control in corporate networks and using power indices in their methods also included floats of small, undefined shareholders in their considerations; see [3,9]. As this paper aims to study a general abstract model of mutual shareholding networks, we try to incorporate this argument into our approach to aggregated power indices. We propose two ways to consider the float: an approach that treats the float as an ocean of an infinite number of unknown shareholders, with each of them holding a small fraction of the shares (Section 3.5), and a fuzzy approach (Section 4).

### 3.2. Motivation and Illustration

We regard an example of a corporate shareholding network that deals with the Speiser and Baker case and which was considered in [10]. In this case, the corporate structure is displayed as a directed graph; see Figure 1. In this network, there are six players, three of which are companies. Namely, three companies are Medallion (1), HealthMed (3), and HealthChem (4). Speiser (2), Baker (5), and others (6) are investors, i.e., firms that are not controlled by any coalitions of firms. For this case, we consider a simple majority, i.e., the majority quota is 50% plus one share.

Our example of a network comprises a number of important features. Namely, we observe a cycle ownership structure (loop) for companies 1, 3, and 4. This network structure is complete in the sense that 100% of each company is controlled by other firms in the network. There is no null player. Player 6 (others) can also be regarded as a float of small investors, i.e., firms that hold a small stock of shares, typically less than 5 percent of the outstanding shares; see Section 3.5 and Section 4.

If we take into consideration only direct ownership in our example, then we can find the minimal winning coalitions for each company. Thus, in company 1, there is only one minimal winning coalition {4}, as company 4 has 100% of the voting rights in company 1. Then, in company 3, there are three minimal winning coalitions of firms that guarantee a simple majority: {1, 2}, {1, 5}, and {2, 5}. In company 4, we have three minimal winning coalitions as well: {2, 3}, {3, 6}, and {2, 5, 6}. Recall that the knowledge of the set of minimally winning coalitions is sufficient to determine a simple game unequivocally and construct the sets of winning coalitions and null player-free winning coalitions (*W*, *W^n−^*) as well (see Section 2.1).

### 3.3. The Karos and Peters Index in the Example

In order to calculate the Φ index in our example (Section 3.2), we first need to incorporate the indirect control in the model of the mutual control structure of the Speiser-Baker network, i.e., to make the mutual structure invariant—*C*. To represent such relations, we define a simple game structure $\left(v_{1}^{C},\;v_{2}^{C},\;v_{3}^{C},\;v_{4}^{C},\;v_{5}^{C},\;v_{6}^{C}\right)$. As firms 2, 5, and 6 are pure investors without shareholders, the games related to them are null, i.e., assign zero to all coalitions. The other simple games $v_{1}^{C},\;v_{3}^{C},\;v_{4}^{C}$ are defined by the sets of minimal winning coalitions. In order to find these sets, we start with direct ownerships and adequate sets of minimal winning coalitions, which are given at the end of Section 3.2. Then, we use the procedure of constructing an invariant mutual control structure—discussed in detail in [61]—which consists of a finite number of steps of elementary substitutions of controlled players by coalitions that control them. For example, company 1 is controlled by company 4, which in turn is controlled by a minimal winning coalition of firms 2 and 3. Thus, we need to add to ${W^{m}(v}_{1}^{C})$ {2, 3}. Repeating such elementary substitutions, at some point, we obtain some sets of minimal winning coalitions that are invariant under such further substitutions. In our example, we obtain the following set of minimal winning coalitions: $W^{m}\left(v_{1}^{C}\right)$ = {{4}, {1, 2}, {2, 3}, {2, 5}, {3, 6}, and {1, 5, 6}}, $W^{m}\left(v_{3}^{C}\right)$ = {{1, 2}, {1, 5}, {2, 3}, {2, 4}, {2, 5}, {4, 5}, and {3, 5, 6}}, and $W^{m}\left(v_{4}^{C}\right)$ = {{1, 2}, {2, 3}, {2, 4}, {2, 5}, {3, 6}, {1, 5, 6}, and {4, 5, 6}}. Note that we also use these sets of minimal winning coalitions to calculate the aggregated indices in Section 3.4. 

Now, let us calculate the Φ index in our example. Table 1 gives the Shapley and Shubik power indices for all firms in the Baker and Spacer case calculated in companies 1, 3, and 4. The indirect control power of all firms in companies 2, 5, and 6 is null, as these companies are not controlled by any firm. They are regarded as pure investors.

Considering the results in Table 1 and Formula (1), we obtain Φ =(−45/60, 58/60, −40/60, −22/60, 29/60, 20/60) = (−0.75, 0.9667, −0.6667, −0.3667, 0.4833, 0.3333). Firms 2 and 5 both have 45% of the stocks of firm 3. However, firm 5 has a 1.5% lower share of stocks in company 4 than firm 2. This results that in the whole shareholder network, the difference in power, calculated according to the Φ index, is greater and Φ_2_(*C*) *=* 2 Φ_5_(*C*).

### 3.4. Aggregated Power Indices a Generalization of the Karos and Peters Approach

In this section, we introduce the aggregated indices and calculate them in the example. Then, the results are discussed in Section 5. 

We provide a table with partial calculations for each aggregated index so that a reader can see how it works. Precisely, each table shows values of respective power indices in simple games corresponding to companies 1, 3, and 4 ($v_{i}^{C}$, *i* = 1, 3, 4). As was mentioned in Section 3.3, values of the indirect control power of all firms in simple games 2, 5, and 6 are null. Therefore, to avoid repetition, in each table with partial calculation, we omit this information. Such information appears only in Table 1 when calculating the Φ index.

#### 3.4.1. The Aggregated Banzhaf Index

For every firm *i* in the network and mutual structure *C*, the aggregated Banzhaf *Aβ* index is defined as follows:

(2) $$A\beta_{i}(C)=\sum_{k\in N}\beta_{i}(v_{k}^{C})-v_{k}^{C}(N)$$

Table 2 shows the results of calculation *β* [49] in companies *i* = 1, 3, and 4. Note that for calculating this index, we need the criticality of each player *j*—expressed by $\eta_{j}\left(v_{i}\right)$—in each simple game *i*. However, knowing the set of minimal winning coalitions for each company is not difficult to obtain.

Considering the results in Table 2 and Formula (2), we obtain *Aβ* = (−15729/20976, 21023/20976, −13789/20976, −10209/20976, 10681/20976, 8023/20976) = (−0.7499, 1.0022, −0.6574, −0.4867, 0.5092, 0.3825). So, according to the aggregated Banzhaf index, the control power of firm 2 (Speiser) is almost twice that of firm 5 (Baker).

#### 3.4.2. The Aggregated Solidarity Index

For every firm *i* in the network and mutual structure *C*, the aggregated solidarity *Aψ* index is defined as follows:

(3) $$A\psi_{i}(C)=\sum_{k\in N}\psi_{i}(v_{k}^{C})-v_{k}^{C}(N)$$

Table 3 shows the results of the calculation of the solidarity index in companies *i* = 1, 3, and 4. Note that for calculating this index, we need to know *Cr*(*S*) for each winning coalition containing player *j*, i.e., the number of critical players in *S,* in the game $v_{i}^{C}$. So, first, it is necessary to find $W_{j}\left(v_{i}^{C}\right)$, starting from the sets of minimal winning coalitions given in Section 3.3.

Considering the results in Table 3 and Formula (3), we obtain *Aψ* = (−1037/1800, 1129/1800, −92/1800, −52/1800, 871/1800, 781/1800) = (−0.5761, 0.6272, −0.5511, −0.4178, 0.4839, 0.4339).

#### 3.4.3. The Aggregated Deegan and Packel Index

For every firm *i* in the network and mutual structure *C*, the aggregated Deegan and Packel *AΔ* index is defined as follows:

(4) $$A\Delta_{i}(C)=\sum_{k\in N}\Delta_{i}(v_{k}^{C})-v_{k}^{C}(N)$$

Table 4 shows the results of the calculation of the *Δ* index in simple games $v_{1}^{C},\;v_{3}^{C},\;v_{4}^{C}$. *Δ* bases on minimal winning coalitions, so knowing the sets $W^{m}\left(v_{i}^{C}\right)$, *i* = 1, 3, 4—given in Section 3.3—the calculations for this index are rather simple.

Considering the results in Table 4 and Formula (4), we obtain *AΔ* = (−151/252, 207/252, −144/252, −144/252, 143/252, 89/252) = (−0.599206349, 0.821428571, −0.571428571, −0.571428571, 0.567460317, 0.353174603).

#### 3.4.4. The Aggregated Holler Index

For every firm *i* in the network and mutual structure *C*, the aggregated Holler index *Ah* is defined as follows:

(5) $$Ah_{i}(C)=\sum_{k\in N}h_{i}(v_{k}^{C})-v_{k}^{C}(N)$$

Note that Bertini, Mercik, and Stach (2023) [17] introduced this index recently and called it the *iPGI* index, where “*i*” refers to indirect control. To be in line with the other nine aggregated newly introduced indices in this paper, we denote the aggregated Holler index by *Ah*.

Let us calculate the *Ah* index in our Example. First, we need to calculate the *h* index in the games corresponding to companies 1, 3, and 4 having $W^{m}\left(v_{i}^{C}\right)$, *i* = 1, 3, and 4, respectively. The adequate result of the calculation is shown in Table 5. Note that *h*, like *Δ*, is based on minimal winning coalitions, and we only need to calculate the membership of each player in all minimal winning coalitions for a particular simple game. 

Considering the results in Table 5 and Formula (5), we obtain *Ah* = (−138/240, 184/240, −138/240, −158/240, 149/240, 101/240) = (−0.5750, 0.7667, −0.5750, −0.6583, 0.6208, 0.4208).

#### 3.4.5. The Aggregated Shift Index

For every firm *i* in the network and mutual structure *C*, the aggregated Shift *As* index is defined as follows:

(6) $$As_{i}(C)=\sum_{k\in N}s_{i}(v_{k}^{C})-v_{k}^{C}(N)$$

Let us calculate the *As* index in our example. First, we need to calculate the Shift index in the games corresponding to companies 1, 3, and 4. The calculation of the *s* index is simple when you have the set of shift minimal winning coalitions. Finding the shift minimal winning coalitions requires using the desirable relationship, which is not so simple anymore. For this reason, we show detailed consideration of how to perform it in the example.

Let us regard only direct ownership. In company 1, there is only one minimal winning coalition {4}, so it is a shift minimal winning coalition as well.

In company 3, there are three minimal winning coalitions: of firms that guarantee a simple majority: {1, 2}, {1, 5}, and {2, 5}. Although firm 2 has more voting rights in company 3 than company 1 (45% versus 10%), both firms are equally desirable in the simple game that refers to company 1 with a simple majority. Indeed, there is no coalition *T* such that i,j∉T, T∪{i}∈W and T∪{j}∉W. In this game, there is only one coalition without firms 1 and 2: coalition {5}. If we add firm 1 or 2 to {5}, we obtain, in both cases, winning coalitions. Next, there is only one non-empty coalition that does not contain firms 2 and 5: coalition {1}. If we add firm 2 to {1}, we obtain a winning coalition. If we add {5} to {1}, we obtain a winning coalition as well. Of course, the empty coalition does not contain any firm. However, no singular coalition is winning. For this reason, we do not consider a single empty coalition here. So, firms 2 and 5 are equally desirable. From another point of view, both firms have the same number of voting rights in company 3—45%. So, consequently, the set of shift minimal winning coalitions is equal to the set of the minimal winning coalitions, which is given as follows: {{1, 2}, {1, 5}, and {2, 5}}. 

In company 4, we have three minimal winning coalitions: {2, 3}, {3, 6}, and {2, 5, 6}, which are also shift minimal winning coalitions. Let us check this. In this company, firm 6 has more voting rights than firm 2. However, both firms are equally desirable. There is no coalition T such that {2}∉T and {6}∉T, ($T\cup \left\{2\right\}\in W$ and $T\cup \left\{6\right\}\notin W$), or (T∪{2}∉W and T∪{6}∈W). In this game, there are only three coalitions without firms 2 and 6: {3}, {5}, and {3, 5}. If we add {2} to {3}, we obtain a winning coalition {2, 3}. If we add {6} to {3}, we obtain a winning coalition {3, 6} as well. If we add {2} to {5}, we obtain a losing coalition {2, 5}. If we add {6} to {5}, we obtain a losing coalition as well. Then, if we add {2} to {3, 5}, we obtain a winning coalition {2, 3, 5}. If we add {6} to {3, 5}, we obtain a winning coalition as well ({3, 5, 6}). So, firms 2 and 6 are equally desirable. Let us consider firms 2 and 3. We have three not empty coalitions without these firms: {5}, {6}, and {5, 6}. Adding {2} to {5} we have a losing coalition; adding {3} to {5} we have a losing coalition as well. Adding {2} to {6} we have losing coalition; adding {3} to {6} we have winning coalition. Adding {2} to {5, 6} we have a winning coalition and adding {3} to {5, 6} we have a winning coalition as well. So, firm 3 is more desirable than firm 2, which is equally desirable as firm 6. Regarding firms 2 and 5, what can we say? We have three coalitions without these firms: {3}, {6}, and {3, 6}. If we add firm 2 to {3}, we obtain a winning coalition. If we add firm 5 to {3}, we obtain a losing coalition. If we add firm 2 or firms 5 or {6}, in both cases, we obtain a losing coalition. Coalition {3, 6} is winning, so adding other players does not change the winning status of the coalition. So, firm 2 is more desirable than firm 5. Thus, 3≻2∼6≻5. In coalition {2, 5, 6} we cannot exchange any player, as firm {3} is more desirable for all members of {2, 5, 6}. As a consequence, we proved that in company 4, the set of shift minimal winning coalitions consists of {2, 3}, {3, 6}, and {2, 5, 6}.

Considering direct and indirect control, we have the following sets of minimal winning coalitions in companies 1, 3, 4: $W^{m}\left(v_{1}^{C}\right)$ = {{4}, {1, 2}, {2, 3}, {2, 5}, {3, 6}, and {1, 5, 6}}, $W^{m}\left(v_{3}^{C}\right)$ = {{1, 2}, {1, 5}, {2, 3}, {2, 4}, {2, 5}, {4, 5}, and {3, 5, 6}}, and $W^{m}\left(v_{4}^{C}\right)$ = {{1, 2}, {2, 3}, {2, 4}, {2, 5}, {3, 6}, {1, 5, 6}, and {4, 5, 6}}; see Section 3.3. Similarly, as in the case of direct control, we apply the desirable relationship to find the shift in minimal winning coalitions for “indirect control” games. 

Company 1 and firm 4 are more desirable than all the others. Adding firm 4 to an empty coalition, we obtain a winning coalition. Adding a player other than 4 to an empty coalition does not result in a winning coalition. Let us regard firms 1 and 2. We have 2^4^ different coalitions without firms 1 and 2. However, we are interested only in coalitions without firm 4. Having firm 4 as a member makes the coalition win. So, we are not interested in these kinds of coalitions, as they do not change the winning status by adding firms 1 or 2. So, in practice, we have 2^3^ coalitions to consider: {3}, {5}, {6}, {3, 5}, {3, 6}, {5, 6}, and {3, 5, 6}. Among these coalitions, we have two minimal winning coalitions, which also are not interesting to us ({3, 6} and {3, 5, 6}). So, the remaining coalitions are {3}, {5}, {6}, {3, 5}, and {5, 6}. Only adding firm 1 to {5, 6} results in a winning coalition. A union of {1} and each S∈{{3}, {5}, {6}, and {3, 5}} results in a losing coalition, whereas the union of firm 2 with only {6} results in a losing coalition. In the rest of the cases, a union with {2} results in a winning coalition. Thus, firm 2 is more desirable than firm 1 (2≻1). Repeating this reasoning for the remaining pairs of firms, we obtain the following picture of the preordering in company 1 by the desirability relation:

4≻i, i∈{1,2,3,5,6}, 2,3≻1∼5, 2,3≻6

From the above, we have that firm 4 dominates all other firms; firms 2 and 3 are incomparable, but they dominate 1, 5, and 6. Firms 1 and 5 are equivalent. Firms 5 and 6 and firms 1 and 6 are incomparable. These are all the relations according to the desirability and strict desirability. Now, {2, 3} can be replaced by {2, 5}, for example, since the last is winning and 5 is weaker than 3. The only minimal winning coalition which is not shift-minimal is {2, 3}. This game is not complete, as some of the firms are not comparable. Thus, $W^{sm}\left(v_{1}^{C}\right)$ = {{4}, {1, 2}, {2, 5}, {3, 6}, and {1, 5, 6}}.

Similarly, considering indirect ownership and desirability relationship, we can find pre-orderings and the sets of shift minimal winning coalitions in companies 3 and 4. For company 3, we have the following preordering of the firms: 2≻5≻1∼4≻3≻6 and $W^{sm}\left(v_{3}^{C}\right)$= {{1, 5}, {2, 3}, {4, 5}, and {3, 5, 6}}. For company 4, we have 2,3≻5≺4∼1, 2≻6 and $W^{sm}\left(v_{4}^{C}\right)$= {{1, 2}, {2, 4}, {3, 6}, {1, 5, 6}, and {4, 5, 6}}.

The results of calculations of the *s* index in companies 1, 3, and 4 are shown in Table 6.

Considering the results in Table 6 and Formula (6), we obtain *As* = (−94/180, 86/180, −107/180, −112/180, 126/180, 101/180) = (−0.52222, 0.47778, −0.59444, −0.62222, 0.70000, 0.56111).

#### 3.4.6. The Aggregated Shift Deegan–Packel Index

For every firm *i* in the network and mutual structure *C*, the aggregated Shift Deegan–Packel *Aµ* index is defined as follows:

(7) $$A\mu_{i}\left(C\right)=\sum_{k\in N}\mu_{i}\left(v_{k}^{C}\right)\;-v_{k}^{C}\left(N\right)$$

Calculation of this index, like the *As* index (Section 3.4.5), requires finding sets of shift minimal winning coalitions in simple games relating to companies 1, 3, and 4. Since such sets have already been found for the *As* index (see Section 3.4.5), we limit ourselves to presenting the results of calculations of the *µ* indices in games $v_{k}^{C},\;k=1,3,4$, in our example; see Table 7.

In Table 7 and Formula (7), we have *Aµ* = (−65/120, 63/120, −71/120, −61/120, 76/120, 58/120) = (−0.5417, 0.5250, −0.5917, −0.5083, 0.6333, 0.4833).

#### 3.4.7. The Aggregated *PI* Index

For every firm *i* in the network and mutual structure *C*, the aggregated Felsenthal winning coalitions least size *API* index is defined as follows:

(8) $$API_{i}(C)=\sum_{k\in N}PI_{i}(v_{k}^{C})-v_{k}^{C}(N)$$

In our example, in the game corresponding to company 1, we have only one winning coalition of the least size: {4}. So, the *PI* index assigns a total power of 1 to company 4; see Table 8.

In company 3, the set of winning coalitions of the least size consists of six coalitions: {1, 2}, {1, 5}, {2, 3}, {2, 4}, {2, 5}, and {4, 5}. Among these coalitions, none contain firm 6. So consequently, firm 6 becomes a null player according to the *PI* index; see Table 8.

In company 4, we have five winning coalitions of the least size: {1, 2}, {2, 3}, {2, 4}, {2, 5}, and {3, 6}. The power assigned to firms 1, 4, 5, and 6, by the *PI* index is the same because each of these firms belongs to only one winning coalition of the least size; see Table 8.

Considering the results in Table 8 and Formula (8), we obtain *API* = (−44/60, 44/60, −43/60, 16/60, 21/60, 6/60) = (−0.7333, 0.7333, −0.7167, 0.2667, 0.35, 0.10). As for the aggregated Banzhaf index, this aggregated index, which is based on the winning coalitions of the least size, also gives much more power to firm 2 than firm 5—more than double that of firm 2.

#### 3.4.8. The Aggregated *f^n^*^−^ Power Index

For every firm *i* in the network and mutual structure *C*, the aggregated null player free winning coalition *Af^n−^* index is defined as follows:

(9) $$Af_{i}^{n-}(C)=\sum_{k\in N}f_{i}^{n-}(v_{k}^{C})-v_{k}^{C}(N)$$

Table 9 shows the results of calculation *f^n−^* in the example. Note that in the example, the none firm is a null firm. So, in all simple games relative to companies 1, 3, and 4, the sets of null player-free winning coalitions are equal to the sets of all winning coalitions. In the game $v_{1}^{C}$, there are six minimal winning coalitions—see Section 3.2—and fifty-one winning coalitions. Then, $\left|W^{m}\left(v_{3}^{C}\right)\right|=\left|W^{m}\left(v_{4}^{C}\right)\right|=7$, $\left|W\left(v_{3}^{C}\right)\right|=43$, and $\left|W\left(v_{4}^{C}\right)\right|=41$.

Considering the results in Table 9 and Formula (9), we obtain *Af^n−^* = (−591686/1078956, 653076/1078956, −571724/1078956, −531744/1078956, 539953/1078956, 502125/1078956) = (−0.5484, 0.6053, −0.5299, −0.4928, 0.5004, 0.4654).

#### 3.4.9. The Aggregated *g^n^*^−^ Power Index

For every firm *i* in the network and mutual structure *C*, the aggregated null player free winning coalition *Ag^n−^* index is defined as follows:

(10) $$Ag_{i}^{n-}(C)=\sum_{k\in N}g_{i}^{n-}(v_{k}^{C})-v_{k}^{C}(N)$$

Table 10 shows the results of the calculation *g^n−^* in the example. Like for the previous index—*f^n−^;* see Section 3.4.8, we first need to find all winning coalitions in each game $v_{i}^{C}$, *i* = 1, 3, 4, and then $W_{j}^{n-}\left(v_{i}^{C}\right)$ for all players *j*. As already mentioned in Section 3.4.8, $W_{j}^{n-}\left(v_{i}^{C}\right)=W\left(v_{i}^{C}\right)$. 

Considering the results in Table 10 and Formula (10), we obtain *Ag* = (−514474/960792, 553290/960792, −502137/960792, −486544/960792, 484459/960792, 465406/960792) = (−0.5355, 0.5759, −0.5226, −0.5064, 0.5042, 0.4844).

#### 3.4.10. The Aggregated Johnston Power Index

For every firm *i* in the network and mutual structure *C*, the aggregated null player free winning coalition *Ag^n−^* index is defined as follows:

(11) $$A\gamma_{i}(C)=\sum_{k\in N}\gamma_{i}(v_{k}^{C})-v_{k}^{C}(N)$$

In order to calculate this index, it is necessary to find all vulnerable coalitions in the simple games, which refer to companies 1, 3, and 4, and consider the direct and indirect control. Having the sets of minimal winning coalitions, we can find 28 vulnerable coalitions in the game $v_{1}^{C}$, 30 in $v_{3}^{C}$, and 31 in $v_{4}^{C}$. Then, we can calculate the Johnston [59] index in each company; see Table 11.

Considering the results in Table 11 and Formula (11), we obtain *Aγ* = (−64530/78120, 91047/78120, −56198/78120, −31980/78120, 36088/78120, 25573/78120) = (−0.8260, 1.1654, −0.7195, −0.4094, 0.4619, 0.3274).

### 3.5. The Float in Aggregated Power Indices

In this section, we present a simple approach for incorporating the float, i.e., the set of unidentified small shareholders, into the framework of aggregated power indices.

It is common to model the float as a random variable that takes values between 0 and 1, i.e., the random variable represents the fraction of the float voting “yes” or 1. Hence, the distribution of this random variable reflects the voting behavior of the float and it allows for different possibilities to model the float. Various models have been introduced in the literature, many of them involving Monte Carlo simulations; see Levy (2011) [62] for a detailed discussion.

Our float model is based on the following result for the Banzhaf index for weighted voting games by Dubey and Shapley (1979) [22]. We assume an oceanic float, i.e., an infinite number of unknown shareholders with each of them holding a vanishingly small fraction of the shares of a company *k* and each of them voting either 0 or 1 with probability *p* = 0.5. In that case, the Banzhaf indices of the *m* known shareholders of company *k* in the weighted voting game for direct control of that company can be approximated by the Banzhaf indices of the modified weighted voting game:

$$\left[q-0.5fl_{k};w_{\left(1,k\right)},\ldots ,w_{\left(m,k\right)}\right]$$

i.e., for a weighted game specified by the quota $\tilde{q}=q-0.5fl_{k}$ and the weights of the *m* are known shareholders, where $fl_{k}$ denotes the total weight of the float. This model was affirmed in empirical studies by Leech (2013) [63].

We propose to use the modified game as a heuristic for aggregated power indices, i.e., we determine the minimal winning coalitions in direct control first from the modified game and then proceed with the minimal winning coalitions in indirect control in order to determine the simple game $v_{k}^{C}$. We note that our heuristic is accurate under the assumption that the float does not vote or that its votes are split equally between “yes” (1) and “no” (0). For our example, our model implies that we no longer interpret player 6 (others) as a monolithic block. According to our model, player 3 now exerts complete direct control over player 4. Considering direct and indirect control, the sets of minimal winning coalitions change as follows. For company 1 we have {{3}, {4}, {1, 2}, {1, 5}, and {2, 5}}. For company 3 we have {{1, 2}, {1, 5}, {2, 3}, {2, 4}, {2, 5}, {3, 5}, and {4, 5}}. Finally, for Company 4 we obtain {{3}, {1, 2}, {1, 5}, {2, 4}, {2, 5}, and {4, 5}}.

We obtain the following results for our aggregated Banzhaf index with a float; see Table 12.

The corresponding aggregated power indices are (−454/693, 505/693, −201/693, −355/693, 505/693) = (−0.6551, 0.7287, −0.2900, −0.5123, 0.7287).

For our aggregated Shapley and Shubik index (Φ) with a float, the picture looks as follows; see Table 13.

The corresponding aggregated power indices Φ are (−42/60, 38/60, −7/60, −27/60, 38/60) = (−0.7000, 0.6333, −0.1167, −0.4500, 0.6333).

## 4. The Karos and Peters Approach with a Fuzzy Float

### 4.1. Basic Notions on Fuzzy Set Theory

In 1965, Zadeh proposed his concept of possibility theory [64]. We will present the basic notions of this theory. First, we will present the concept of a fuzzy number. Let $\tilde{X}$ be a single-valued number whose value is not precisely known. The membership function for X is a normal, quasi concave, and upper semi-continuous function $\mu_{X}:R\to \left[0,1\right]$; see [65,66]. The value $\mu_{X}\left(x\right)$ for x∈R denotes the possibility of the event that the fuzzy number $\tilde{X}$ takes the value of x. We denote this as follows:

(12) $$\mu \left(x\right)=Pos\left(\tilde{X}=x\right)$$

For a given fuzzy number X and a given λ, the λ-level is defined to be the closed interval ${\left[\tilde{X}\right]}_{\lambda }=\left\{x:\mu \left(x\right)\geq \lambda \right\}=\left[\underline{x}\left(\lambda \right),\bar{x}\left(\lambda \right)\right].$

Dubois and Prade (1978) [67] introduced the following useful definition of the L-R class of fuzzy variables. The fuzzy number $\tilde{X}$ is called an L-R type fuzzy number when its membership function takes the following form:

(13) $$\mu_{X}\left(x\right)=\left\{\begin{array}{c} \begin{array}{ccc} L\left(\frac{\underline{m}-x}{\alpha }\right) & for & \underline{m}-\alpha <x<\underline{m}\\ 1 & for & \underline{m}\leq x\leq \bar{m}\\ R\left(\frac{x-\bar{m}}{\beta }\right) & for & \bar{m}<x<\bar{m}+\beta \\ 0 & \mathrm{otherwise} & \end{array} \end{array}\right.$$

where $L\left(x\right)$ and $R\left(x\right)$ are continuous non-increasing functions, x, α, β>0, $\left[\underline{m},\;\bar{m}\right]$ are the most possible values, $\alpha =\underline{m}-\underline{x}\left(0\right)$ is the left spread, and $\beta =\bar{x}\left(0\right)-\bar{m}$ is the right spread of the fuzzy number. 

The functions $L\left(x\right)$ and $R\left(x\right)$ are called the left and the right spread functions, respectively. The most commonly used spread functions are $max\left\{0,1-x^{p}\right\}$ and $exp\left(-x^{p}\right)$, $x\in \left[\left.0,+\infty \right)\right.$, p≥1. An interval fuzzy number for which $L\left(x\right)$=$R\left(x\right)=max\left\{0,1-x\right\}$ and $\underline{m}=\bar{m}=m$ is called a triangular fuzzy number $\tilde{X}$ and will be denoted by $\left(m_{X},\alpha_{X},\beta_{X}\right)$. 

Let $\tilde{X},\tilde{Y}$ be two fuzzy numbers with membership functions, respectively, $\mu_{X}\left(x\right)\;\mathrm{and}\;\mu_{Y}\left(y\right)$, and let z=f(x,y). Then, according to the extension principle of Zadeh [64,68], the function of belonging to the fuzzy set $\tilde{Z}=f\left(\tilde{X},\tilde{Y}\right)$ takes the following form:

(14) $$\mu_{Z}\left(z\right)=\sup_{z=f(x,y)}\left(\min\left(\mu_{X}\left(x\right),\mu_{Y}\left(y\right)\right)\right)$$

According to the fuzzy logic proposed by Zadeh (1965) [64], the membership function of the logic operator $\tilde{X}$ and $\tilde{Y}$ takes the following form:

(15) $$\mu_{\tilde{X}\mathrm{and}\tilde{Y}}\left(x\right)=\min(\mu_{X}\left(x\right),\mu_{Y}\left(x\right))$$

If we want to compare two fuzzy sets, that is, to determine the possibility that realization $\tilde{X}$ is not less (or greater respectively) than the realization $\tilde{Y}$, then we can use the index proposed by Dubois and Prade (1988) [65]:

(16) $$Pos\left(\tilde{X}\geq \tilde{Y}\right)=\underset{x}{\sup}\min\left(\min\left(\mu_{X}\left(x\right),\mu_{Y}\left(y\right)\right)\right)\;Pos\left(\tilde{X}>\tilde{Y}\right)=\underset{x}{\sup}\underset{y\geq x}{\inf}\left(\min\left(\mu_{X}\left(x\right),1-\mu_{Y}\left(y\right)\right)\right)$$

If $\tilde{X}$ is a triangular fuzzy variable $\tilde{X}=\left(x,l_{X},r_{X}\right)$, then its expected value is equal to (Carlsson and Füllér (2001) [69] and Chanas and Nowakowski (1988) [70]):

(17) $$E(\tilde{X})=x+\frac{r_{X}-l_{X}}{4}$$

In 2011, Zadeh introduced the concept of a Z-fuzzy number [71]. A Z-fuzzy number is an ordered pair of fuzzy numbers *Z* = (A,B). A Z-fuzzy number is associated with a real-valued uncertain variable, X, with the first component, A, playing the role of a fuzzy restriction, R(X), on the values which X can take, written as X is A, where A is a fuzzy set and B is a measure of reliability (certainty) of the A. In the literature, one can find a list of fuzzy triangular numbers, each corresponding to a linguistic (reliability-related) expression, such as sure, usually, likely, etc. An example of the “dictionary” for the values of B can be found in Table 14.

In the literature, there have been several proposals of arithmetic operations on *Z*-fuzzy numbers, e.g., [73,74,75,76], which differed in the procedure to encapsulate the information given by the ordered pair (*A*, *B*) in a simplified form (as a classical fuzzy number or in a defuzzified form, as a crisp number), in order to make comparisons among *Z*-fuzzy numbers. Defuzzification is a method often used in practice in order to summarize the information conveyed by a fuzzy number of any type. Of course, defuzzification always entails a loss of information. 

The crisp equivalent of the second part *B* (reliability) of Z-number is obtained as a center of gravity method:

(18) $$\theta =\frac{\int_{R}^{}x\mu_{B}\left(x\right)dx}{\int_{R}^{}\mu_{B}\left(x\right)dx}$$

If B is a triangular fuzzy number $B=\left(m_{B},\alpha_{B},\beta_{B}\right)$, then its center of gravity is equal to:

(19) $$\theta =m_{B}+\frac{\beta_{B}-\alpha_{B}}{3}$$

A *Z*-fuzzy number can be converted into the following classical fuzzy number [74,75]:

(20) $$Z{}^{\prime }=\sqrt{\theta }Z^{\theta }=\left(\sqrt{\theta }m_{A},{\sqrt{\theta }\alpha }_{A},\sqrt{\theta }\beta_{A}\right)$$

Hence, it follows that the expected value of the converted *Z*-fuzzy number is equal to:

(21) $$E\left(Z{}^{\prime }\right)=\sqrt{\theta }E\left(A\right)$$

### 4.2. The Karos and Peters Approach to a Fuzzy Float

Let us now assume that in complex corporate shareholding structures, the behavior of “float shareholders,” i.e., (float shareholders’ weights), is expressed by the *Z*-fuzzy number *Z* = (*A*, *B*). Thus, we assume that our knowledge about the behavior of those anonymous by definition and known only as an aggregate summing up the number of shares held is purely expert. Depending on our knowledge (e.g., resulting from historical behavior), we can assess in a fuzzy way what part of this aggregate we should consider when assessing the possibility of a given coalition. For example, it occasionally happens that a representative of small shareholders is registered in a meeting in the decision-making body of a given company (for example, the general meeting of shareholders) who, based on granted powers of attorney, behave like a single shareholder with a designated number of shares. Then, the subject of the analysis is to determine (in our case, in a fuzzy way) both how often this can happen and what package of such authorizations we can deal with.

To find the Karos and Peters index [1] for complex corporate shareholding structures in the case when the behavior of “float shareholders” is defined by *Z*-fuzzy numbers, we propose Algorithm 1 for defuzzification fuzzy weights.
**Algorithm 1** Defuzzification fuzzy weightsStep 1. Transform float shareholders’ weights expressed by the *Z*-fuzzy number *Z* = (*A*, *B*) into classical fuzzy numbers *Z*′ using the dictionary for the values of the second component B and Formulas (18)–(20). Step 2. Find a crisp equivalent of float shareholders’ weights, i.e., expected value *E*(*Z*′), using Formula (21).

### 4.3. Karos and Peters Index for the Fuzzy Float for the Corporate Shareholding Network Which Deals with the Speiser and Baker Case

Let us consider the corporate shareholding network which deals with the Speiser and Baker case presented in Figure 1. Let us further assume that the weight (possible votes) of player 6 (others) is given as a triangular *Z*-fuzzy number in the form (*A*, *B*) = ((0, 0, 40), likely), where (0, 0, 40) is a classical triangular fuzzy number and B is a measure of reliability (certainty) of the A. On the basis of Algorithm 1, we can transform the *Z*-fuzzy number ((0, 0, 40), likely) = ((0, 0, 40), (0,6, 0.1, 0.1)) into a classical triangular fuzzy number (0, 0, 30.98). The expected value of possible votes (crisp weight after defuzzification) of firm 6 (others) is equal to 7.74597.

When we take into consideration only direct ownership in the corporate shareholding (Figure 1) in the fuzzy case, then we can find the minimal winning coalitions for each company. Thus, in company 1, there is only one expected minimal winning coalition {4}, as company 4 has 100% voting rights in company 1. Then, in company 3, there are expected to be three minimal winning coalitions of firms that guarantee a simple majority: {1, 2}, {1, 5}, and {2, 5}. In company 4, we can expect two minimal winning coalitions: {2, 3} and {3, 5, 6}.

Let us now calculate the Φ index. Considering direct and indirect control, we have the following set of minimal winning coalitions in company 1: {4}, {1, 2}, {2, 3}, {2, 5}, and {3, 5, 6}. Similarly, for company 3, we have the following set of minimal winning coalitions: {1, 2}, {1, 5}, {2, 5}, {2, 4}, {4, 5}, {2, 3}, and {3, 5, 6}. Finally, for company 4, the set of minimal winning coalitions is as follows: {2, 3}, {1, 2}, {2, 5}, {2, 4}, {3, 5, 6}, {1, 5, 6}, and {4, 5, 6}. Table 15 gives the Shapley and Shubik power index for all firms in the Baker and Spacer case calculated in companies 1, 3, and 4. The indirect control power of all firms in firms 2, 5, and 6 is null, as these companies are not controlled by any firm. They are regarded as pure investors. 

Based on the results in Table 15 and Formula (1), we obtain Φ =(−45/60, 65/60, −48/60, −20/60, 36/60, 12/60). Φ for firm 2 and firm 5 is greater by 7/60, and for firm 6 is smaller by 8/60 than in the case of the classical game (Section 3.3). 

As mentioned earlier, defuzzification is a method often used in practice in order to summarize the information conveyed by a fuzzy number of any type. Of course, defuzzification always entails a loss of information. 

Table 16 presents fuzzy weights for coalitions that include firm 6. Let us calculate the possibility that a given coalition is the minimal winning coalition using Formula (17). After defuzzification in Step 1 of Algorithm 1, the total classical fuzzy weight of coalition {3, 6} and coalition {3, 5, and 6} are equal to 41.5 + (0, 0, 30.98) = (41.5, 0, 30.98) and 41.4 +8.5 + (0, 0, 30.98) = (50, 0, 30.98), respectively. According to (16) possibility that coalition {3, 6} is minimal winning one is equal to *Pos*(41.5,0,30.98>50) = 0.73. Further possibility that coalition {3, 5, and 6} is a minimal winning coalition is equal to *Pos*($50<\left(50,0,30.98\right)\leq 58.5$) = 0.27; see Formula (16). For the other coalitions that include company 6, the possibility that they are minimal winning coalitions equals 0. Furthermore, according to Formulas (15) and (16), the possibility that company 6 is a null player is equal to $Pos(50>=\tilde{W}\{6\}\;\mathrm{and}\;50>=\tilde{W}\{2,6\}\;\mathrm{and}\;50>=\tilde{W}\{3,6\}\;\mathrm{and}\;50>=\tilde{W}\{5,6\}\;\mathrm{and}\;50>=\tilde{W}\{2,5,6\}\;\mathrm{and}\;50>=\tilde{W}\{3,5,6\})=1.$

Note that in the deterministic case (Section 3.3), when we assume that weight (firm 6) = 40%, coalition {2, 5, and 6} is a minimal winning coalition. In the fuzzy case, when we are unsure about small investors’ behavior, the possibility that coalition {2, 5, and 6} is a minimal winning coalition is equal to 0. Furthermore, the possibility that company 6 (float) is a null player is equal to 1. 

## 5. Discussion, Comparison, and Conclusions

This paper proposes some new measures of indirect control power in complex shareholding structures. These new game theory approaches to measuring indirect control are based on the Karos and Peters [1] method, and they follow the proposal of Stach, Mercik, and Bertini (2023) [17]. Namely, in [17], the authors, instead of using the Shapley and Shubik [2] index in the Karos and Peters framework, proposed the Holler [54,55] index (also called the public good index). We follow this idea, and we apply in the framework of Karos and Peters some power indices substituting the original Shapley and Shubik power index in a modular fashion.

An interesting and new idea is the fuzzy approach to the float and applying it to calculate the control power of all firms in a network by the Karos and Peters method. A measure that considers the float of small shareholders (usually those with lower or equal 5% ownership) seems closer to the real world.

Table 17 compares the newly proposed aggregated indices in the example.

In particular, the ranking of investors is the same for all power indices except the shift indices, *As* and *Aµ*. The *As* an*d Aµ* indices rank investors from most to least powerful, as follows: 5, 6, 2, and 5, 2, 6, respectively. The rest of the indices rank firm 2 as the most powerful, then, followed by Firm 5, and the least powerful is firm 6. This is largely because firm 2 is the most desirable in companies 3 and 4, and consequently belongs to fewer numbers of the shift minimal winning coalitions. Firm 2 has more control power than firm 6 according to *Aµ,* in contrast to the order given by *As*. This is because firm 2 belongs to a greater number of less numerous shift minimal winning coalitions than firm 6, and the *Aµ* index also considers the size of the shift minimal winning coalitions (see Section 3.4.5 and Section 3.4.6).

The aggregated power indices vary a lot in the ranking of companies. Φ, *Aβ*, *Aψ*, *API, AΔ*, *Af^n^*^−^, *Ag^n^*^−^, and *γ* give the same ranking. According to these indices, the least powerful is company 1, followed by company 3, and the most powerful is company 4. Let us add that *AΔ* gives the same control power to company 3 and 4. *Aµ* also classifies company 4 in the first place, but it attributes more power to company 1 than to company 3. This is because, as mentioned above, the size of the shift minimal winning coalition is important for *Aµ*. Participation in less numerous coalitions results in greater control power for *Aµ*. *Ah* and *As* rank the companies differently. The least powerful is company 4 and the most powerful company 1. *Ah* gives the same importance (power) to companies 1 and 3. According to *As*, company 3 has less power than company 1, which is in accordance with *Aµ*. All indices, except *As* and *Aµ,* give company 3 at least as much power as company 1. 

According to our simple heuristic for the float based on the result by Dubey and Shapley [22], company 3 exerts complete direct control over company 4 and indirectly on company 1 as well. Thus, in this model, Φ and *Aβ* rank company 3 in first place, then companies 4 and 1. As for the ranking of firms 2 and 5, they have equal control power. This means that considering the float and its ability to strengthen company 3, firm 2 loses its control power in favor of company 3’s control power.

When we assume nondeterministic (fuzzy) float’s behavior, then according to the expected value of index Φ*,* the least powerful is company 3, followed by company 1, and the most powerful is company 4. It means that index Φ gives company 3 smaller power than company 1, which is opposite to the deterministic version. However, we should underline that possibility of such companies’ power ranking is equal to 0.73. In the fuzzy case, the possibility that company 6 (float) is a null player is equal to 1. 

Considering the exact numbers of power assigned by power indices, we see that indices vary substantially. Namely, sums of power assigned to companies are greater or lower depending on the index. Let us remember that the total power assigned to companies and investors always adds up to 0. Next, *Aγ* assigns the greatest power to firm 2 among all aggregated indices. For only two indices, *Aγ* and *Aβ*, this value is even greater than 1. Moreover, *API* gives the greatest power to company 4 of all power indices.

Turning back to the ranking of the players given by the aggregated indices, we have some new observations. When we consider only direct ownership and weighted games related to stock companies, then, of course, in each game, the rankings given by the Shapley and Shubik, Banzhaf, and Johnston indices must be the same. This is due to two facts: each weighted game is a complete game (see Section 2.1), and in complete games, the mentioned three indices are ordinary equivalent, so their rank the players in an equal way; see Freixas, Marciniak, and Pons (2012) [77]. If we consider indirect control, the games can become non-complete (non-linear). This means that not all players can be comparable by the desirability relationship as in the example presented in Section 3.4.5. If a game is not complete, the rankings of players produced by the Shapley and Shubik, Banzhaf, and Johnston indices are not necessarily the same, which does not happen in the example. By the definitions of aggregated indices, what happens in “direct” games (games based on direct ownership only) influences the “indirect” games (games that take into account indirect ownership). Of course, the aggregated indices calculate the control power of firms in the whole network by summing up the values of respective indices over all companies. Thus, the ranking of players produced by an aggregated index is a consequence of what happens in the whole network considering indirect control. However, knowing that “indirect” games are not complete, it is difficult to expect that, in general, the rankings of the Φ, *Aβ*, and *Aγ* indices will always be the same. In [12,17], the reader can find an example of a theoretical shareholding network for which calculated indices Φ, Aβ, and Aγ produce different rankings of firms.

The Φ index satisfies five axioms; see Section 2.3. If we change the Shapley and Shubik [2] index in the definition of Φ given by Formula (1), it is difficult to expect that all five axioms will be satisfied by the resulting aggregated index. 

Axiom 1—the null player axiom from the Karos and Peters approach—is only satisfied by aggregated indices constructed on the power indices that fulfill adequate null player postulate in simple games.

If we take into consideration an efficient power index in the framework of the Karos and Peters approach, then the appropriate aggregated index preserves axiom 2—the constant sum property—by construction; see Formula (1). All power indices considered by us are efficient; so, all aggregated indices fulfill this axiom.

Karos and Peters treat axioms 1 and 2 as the scaling conditions. Let us cite them: “the null player and constant sum postulates have a considerable impact on the resulting power index, but they can be seen as scaling conditions, which are needed anyway in some form or another” [1] (p. 160).

Obviously, a very natural condition called the anonymity axiom is also satisfied by all aggregated indices, as all considered indices from our three groups (see Section 2.2.2) fulfill this postulate in simple games.

Regarding the fulfillment of axiom 4, we have only one candidate, except of course, the Φ index and the aggregated Solidarity index, as only these indices satisfy the transfer postulate in simple games; see Section 2.2.2 and [52]. Additionally, let us note that this property is one of the properties characterizing this index axiomatically; see Nowak and Radzik (1994) [52]. However, no fulfillment of the transfer property by a particular power index should be seen as an obstacle to using such an index. Let us quote the authors of the Φ index themselves: “We regard the transfer property as a basic axiomatic choice, in the sense that one should drop this condition in order to obtain essentially different power indices” [1] (p. 160).

Axiom 5—the controlled player condition—is satisfied by the aggregated indices based on power indices that fulfill null player property in simple games. This is given by the construction of Formula (1).

Which of the presented indices is the best one? The answer is: it depends. The choice of the method to assess the control power of firms in a mutual shareholding network comes down to decision-maker preferences. The choice may be based on the set of desirable properties satisfied by a particular index or on the convincing normative bargaining model underlying this index. For example, if someone searches for the control power of firms in a network that considers firms’ strength to form minimal winning coalitions, then the group of aggregated minimal winning coalition indices is preferable (*AΔ*, *Ah*, *As*, and *API*). If the local monotonicity property is also important, then the *APL* index is the best choice. So, the solution to the posted question could be a compromise. It means that in searching for information about firms’ control power, we are willing to sacrifice one of the axioms and find an acceptable choice method. The axioms the methods violate can be thought of as cautions: if some axiom is important for a particular information, do not use that method that violates that axiom. For example, power indices based on minimal winning coalitions (the second group of indices in Section 2.2.2) violate axiom 4 (transfer). This implies that aggregated power indices based on minimal winning coalitions should not be used in an analysis for which the transfer property is considered to be important (see postulates 3, 3′, and 3″ in Section 2.2.1).

Considering the further developments of our framework, it is interesting to consider other indices in the scheme proposed by Karos and Peters (2015) [1], such as the minimum sum representation (MSR) index, proportional to the voting weight, introduced by Freixas and Kaniovski (2014) [78]. Note that the MSR index is ordinally equivalent to the Banzhaf [50], Shapley and Shubik [2], and Johnston [59] indices.

One of the promising fields of future research appears to measure the indirect control in complex corporate networks with fuzzy weights and follows the idea of Dubois and Prade (1978) [67] to propose an adequate concept of the power index.

## Figures and Tables

**Table 1:** The σ index is calculated for each player and a simple game is defined for our example.

Simple Game *v_i_*	Player 1	Player 2	Player 3	Player 4	Player 5	Player 6
*i* = 1	4/60	12/60	7/60	27/60	4/60	6/60
*i* = 3	7/60	22/60	4/60	7/60	19/60	1/60
*i* = 4	4/60	24/60	9/60	4/60	6/60	13/60
*i* = 2, 5, 6	0	0	0	0	0	0
Total	15/60	58/60	20/60	38/60	29/60	20/60

**Table 2:** The *β* index is calculated for each player and a simple game is defined for our example.

Game *v_i_*	Player 1	Player 2	Player 3	Player 4	Player 5	Player 6
*i* = 1	3/38	9/38	5/38	13/38	3/38	5/38
*i* = 3	5/46	17/46	3/46	5/46	15/46	1/46
*i* = 4	3/48	19/48	7/48	3/48	5/48	11/48
Total	5247/20976	21023/20976	7187/20976	10767/20976	10681/20976	8023/20976

**Table 3:** The *ψ* index is calculated for each player and a simple game is defined for our example.

Simple Game *v_i_*	Player 1	Player 2	Player 3	Player 4	Player 5	Player 6
*i* = 1	465/3600	615/3600	525/3600	1035/3600	465/3600	495/3600
*i* = 3	558/3600	804/3600	498/3600	558/3600	744/3600	438/3600
*i* = 4	503/3600	839/3600	593/3600	503/3600	533/3600	629/3600
Total	1526/3600	2258/3600	1616/3600	2096/3600	1742/3600	1562/3600

**Table 4:** The *Δ* index is calculated for each player and a simple game is defined for our example.

Simple Game *v_i_*	Player 1	Player 2	Player 3	Player 4	Player 5	Player 6
*i* = 1	5/36	9/36	6/36	6/36	5/36	5/36
*i* = 3	6/42	12/42	5/42	6/42	11/42	2/42
*i* = 4	5/42	12/42	6/42	5/42	7/42	7/42
Total	101/252	207/252	108/252	108/252	143/252	89/252

**Table 5:** The *h* index is calculated for each player and a simple game is defined for our example.

Simple Game *v_i_*	Player 1	Player 2	Player 3	Player 4	Player 5	Player 6
*i* = 1	2/12	3/12	2/12	1/12	2/12	2/12
*i* = 3	2/15	4/15	2/15	2/15	4/15	1/15
*i* = 4	2/16	4/16	2/16	2/16	3/16	3/16
Total	102/240	184/240	102/240	82/240	149/240	101/240

**Table 6:** The *s* index is calculated for each player and a simple game is defined for our example.

Simple Game *v_i_*	Player 1	Player 2	Player 3	Player 4	Player 5	Player 6
*i* = 1	2/10	2/10	1/10	1/10	2/10	2/10
*i* = 3	1/9	1/9	2/9	1/9	3/9	1/9
*i* = 4	2/12	2/12	1/12	2/12	2/12	3/12
Total	86/180	86/180	73/180	68/180	126/180	101/180

**Table 7:** The *µ* index is calculated for each player and a simple game is defined for our example.

Simple Game *v_i_*	Player 1	Player 2	Player 3	Player 4	Player 5	Player 6
*i* = 1	5/30	6/30	3/30	6/30	5/30	5/30
*i* = 3	3/24	3/24	5/24	3/24	8/24	2/24
*i* = 4	5/30	6/30	3/30	5/30	4/30	7/30
Total	55/120	63/120	49/120	59/120	76/120	58/120

**Table 8:** The *PI* index is calculated for each player and a simple game is defined for our example.

Simple Game *v_i_*	Player 1	Player 2	Player 3	Player 4	Player 5	Player 6
*i* = 1	0	0	0	1	0	0
*i* = 3	2/12	4/12	1/12	2/12	3/12	0
*i* = 4	1/10	4/10	2/10	1/10	1/10	1/10
Total	16/60	44/60	17/60	76/60	21/60	6/60

**Table 9:** The *f^n−^* index is calculated for each player and a simple game is defined for our example.

Game *v_i_*	Player 1	Player 2	Player 3	Player 4	Player 5	Player 6
*i* = 1	46/306	54/306	49/306	63/306	46/306	48/306
*i* = 3	27/172	36/172	25/172	27/172	34/172	23/172
*i* = 4	71/492	108/492	81/492	71/492	75/492	86/492
Total	487270/D *	653076/D *	507232/D *	547212/D *	539953/D *	502125/D *

* D = denominator = 1,078,956

**Table 10:** The *g^n−^* index is calculated for each player and a simple game is defined for our example.

Game *v_i_*	Player 1	Player 2	Player 3	Player 4	Player 5	Player 6
*i* = 1	27/172	30/172	28/172	32/172	27/172	28/172
*i* = 3	24/152	30/152	23/152	24/152	29/152	22/152
*i* = 4	22/147	30/147	24/147	22/147	23/147	26/147
Total	446318/D *	553290/D *	458655/D *	474248/D *	484459/D *	465406/D *

* D = denominator = 960,792

**Table 11:** The *γ* index is calculated for each player and a simple game is defined for our example.

Simple Game *v_i_*	Player 1	Player 2	Player 3	Player 4	Player 5	Player 6
*i* = 1	8/168	39/168	18/168	78/168	8/168	17/168
*i* = 3	15/180	78/180	8/180	15/180	62/180	2/180
*i* = 4	8/186	93/186	24/186	8/186	13/186	40/186
Total	13590/D *	91047/D *	21922/D *	46140/D *	36088/D *	25573/D *

* D = denominator = 78,120

**Table 12:** The *β* index for each player and a simple game is defined for our example with a float.

Simple Game *v_i_*	Player 1	Player 2	Player 3	Player 4	Player 5
*i* = 1	1/7	1/7	2/7	2/7	1/7
*i* = 3	1/11	4/11	1/11	1/11	4/11
*i* = 4	1/9	2/9	3/9	1/9	2/9
Total	239/693	505/693	492/693	338/693	505/693

**Table 13:** The Φ index for each player and a simple game is defined for our example with a float.

Simple Game *v_i_*	Player 1	Player 2	Player 3	Player 4	Player 5
*i* = 1	2/20	2/20	7/20	7/20	2/20
*i* = 3	2/20	7/20	2/20	2/20	7/20
*i* = 4	6/60	11/60	26/60	6/60	11/60
Total	18/60	38/60	53/60	33/60	38/60

**Table 14:** An example of the dictionary for the values of the second component *B* of a *Z*-fuzzy number.

*B*	Fuzzy Reliability
Sure	(1, 0, 0.2)
Usually	(0.75, 0.1, 0.1)
Likely	(0.6, 0.1, 0.1)

Source: Azadeh and Kokabi (2016) [72].

**Table 15:** The expected values of the Shapley and Shubik index are calculated for each player and a simple game is defined for our example in the fuzzy case.

Simple Fuzzy Game $v_{i}$	1	2	3	4	5	6
Company 1	4/60	14/60	4/60	29/60	6/60	3/60
Company 3	7/60	22/60	4/60	7/60	19/60	1/60
Company 4	4/60	29/60	4/60	4/60	11/60	8/60
Total	15/60	65/60	12/60	40/60	36/60	12/60

**Table 16:** Total fuzzy weights for coalitions that include firm 6 and the possibility that a given coalition is a minimal winning one in a simple game is defined for our example in the fuzzy case.

Coalition	Total Fuzzy Weight $\tilde{W}$	Possibility That Coalition is Minimal Winning
{6}	(0, 0, 30.98)	0
{2, 6}	(10, 0, 30.98)	0
{3, 6}	(41.5, 0, 30.98)	0.73
{5, 6}	(8.5, 0, 30.98)	0
{2, 5, 6}	(18.5, 0, 30.98)	0
{3, 5, 6}	(50, 0, 30.98)	0.27

**Table 17:** A comparison of power indices in the example.

Index	Player 1	Player 2	Player 3	Player 4	Player 5	Player 6
Φ	−0.7500	0.9667	−0.6667	−0.3667	0.4833	0.3333
*Aβ*	−0.7499	1.0022	−0.6574	−0.4867	0.5092	0.3825
*Aψ*	−0.5761	0.6272	−0.5511	−0.4178	0.4839	0.4339
*A* *Δ*	−0.5992	0.8214	−0.5714	−0.5714	0.5675	0.3532
*Ah*	−0.5750	0.7667	−0.5750	−0.6583	0.6208	0.4208
*As*	−0.5222	0.4778	−0.5944	−0.6222	0.7000	0.5611
*Aµ*	−0.5417	0.5250	−0.5917	−0.5083	0.6333	0.4833
*API*	−0.7333	0.7333	−0.7167	0.2667	0.3500	0.1000
*Af^n−^*	−0.5484	0.6053	−0.5299	−0.4928	0.5004	0.4654
*Ag^n−^*	−0.5355	0.5759	−0.5226	−0.5064	0.5042	0.4844
*Aγ*	−0.8260	1.1654	−0.7195	−0.4094	0.4619	0.3274
Φ with binary float	−0.7000	0.6333	−0.1167	−0.4500	0.6333	–
*Aβ* with binary float	−0.6551	0.7287	−0.2900	−0.5123	0.7287	–
Φ with fuzzy float	−0.7500	1.0833	−0.8000	−0.3333	0.6000	0.2000

## Data Availability

Not applicable.
